# Diet-Derived Circulating Antioxidants and Risk of Digestive System Tumors: A Mendelian Randomization Study

**DOI:** 10.3390/nu14163274

**Published:** 2022-08-10

**Authors:** Linlin Yin, Haihao Yan, Kangdi Chen, Zuhong Ji, Xiuhua Zhang, Guozhong Ji, Bin Zhang

**Affiliations:** 1 Department of Digestive Diseases, The Second Hospital of Nanjing, Nanjing University of Chinese Medicine, Nanjing 210046, China; 2 Medical Center for Digestive Diseases, Second Affiliated Hospital, Nanjing Medical University, Nanjing 210029, China

**Keywords:** digestive system tumors, circulating antioxidants, mendelian randomization

## Abstract

Previous observational case-control studies have shown significant controversy over the impact of dietary intake-related circulating antioxidants on the risk of digestive system tumors. We conducted a two-sample Mendelian randomized (MR) analysis to determine whether there was a significant causal relationship between increased levels of circulating antioxidants and digestive system tumors. Our circulating antioxidants (vitamin C, carotenoids, vitamin A, and vitamin E) were derived from absolute circulating antioxidants and circulating antioxidant metabolites, and their corresponding instrumental variables were screened from published studies. The digestive system tumors we studied included colorectal, gastric, pancreatic, liver, and esophageal cancer, and the corresponding summary GAWS (genome-wide association study) data were obtained from the UK Biobank database. We first evaluated the causal relationship between each tumor and circulating antioxidants and then used meta-analysis to summarize the results of MR analysis of different tumors. No significant associations were noted for genetically predicted circulating antioxidants and higher risk of digestive system tumors in our study. The pooled ORs (odds ratio) are 0.72 (95% CI: 0.46–1.11; β-carotene), 0.93 (95% CI: 0.81–1.08; lycopene), 2.12 (95% CI: 0.31–14.66; retinol), and 0.99 (95% CI: 0.96–1.02; ascorbate) for absolute circulating antioxidants; for circulating antioxidant metabolites, the pooled ORs for digestive system tumors risk per unit increase of antioxidants were 1.29 (95% CI: 0.39–4.28; α-tocopherol), 1.72 (95% CI: 0.85–3.49; γ-tocopherol), 1.05 (95% CI: 0.96–1.14; retinol), and 1.21 (95% CI: 0.97–1.51; ascorbate), respectively. Our study suggested that increased levels of dietary-derived circulating antioxidants did not reduce the risk of digestive system tumors.

## 1. Introduction

Digestive system tumors include colorectal cancer, gastric cancer, esophageal cancer, pancreatic cancer, and liver cancer. Digestive system tumors currently have high morbidity and mortality, with more than 4.8 million new cases and 3.4 million deaths each year [1]. Meanwhile, they are considered to be the most common malignancies leading to cancer-related deaths worldwide and have become one of the most important public health problems [2]. According to age group and world population growth projections, the number of new cases and mortality is expected to increase by 58% and 73% to 7.5 million and 5.6 million, respectively, by 2040 [3].

Epidemiological studies have shown that most digestive system tumors are caused by excessive drinking, smoking, obesity, oxidative stress, Helicobacter pylori infection, and improper diet [4]. Among them, oxidative stress is associated with the occurrence and development of most tumors. Extensive experimental evidence has demonstrated the role of oxidative stress in digestive system tumor initiation, promotion, and progression [5,6,7]. Under oxidative stress, intracellular reactive oxygen species (ROS) levels override the antioxidant defense mechanisms. Excessive reactive oxygen species can damage the mitochondrial and genomic DNA, leading to DNA damage, molecular mutations, and changes in signaling pathways, which are closely related to tumorigenesis and development [8].

As ROS promotes the development of tumors, antioxidants are widely used in gastrointestinal cancers prevention as substances that neutralize reactive oxygen species. Among them, vitamin A (retinol), vitamin E (α-and γ-tocopherol), vitamin C (ascorbate), and carotenoids (β-carotene, lycopene) are the main dietary intake-related antioxidants. Vitamin E and carotenoids are fat-soluble antioxidants, while vitamin C is a water-soluble antioxidant, which can inhibit chemical-induced gastrointestinal neoplasms by scavenging oxygen free radicals and other mechanisms [9]. Meanwhile, vitamin A plays a key role in cell differentiation and inhibition of tumor cell growth [10].

Although many studies have shown the relationship between dietary intake of antioxidants and digestive system tumors, considering most of these studies are derived from case-control studies, the results are obviously controversial [11,12,13]. The case-control study itself has some weaknesses. First, the case-control study makes it difficult to judge the time sequence of exposure factors and diseases, failing to clarify their causal relationship. At the same time, case-control studies are prone to selection bias in the selection of subjects and information bias during the retrospective investigation. However, Mendelian randomization, a method of inferring potential causal relationships, has been widely used to assess the association between risk factors and disease occurrence. At the same time, MR is based on the fact that alleles are randomly assigned from parents to offspring, and fixed genotypes during the formation of the zygote are not affected by the disease, thus avoiding the problem of mixed bias and reverse causality [14].

## 2. Materials and Methods

### 2.1. Genetic Instrumental Variables for Antioxidants

**Figure 1:**
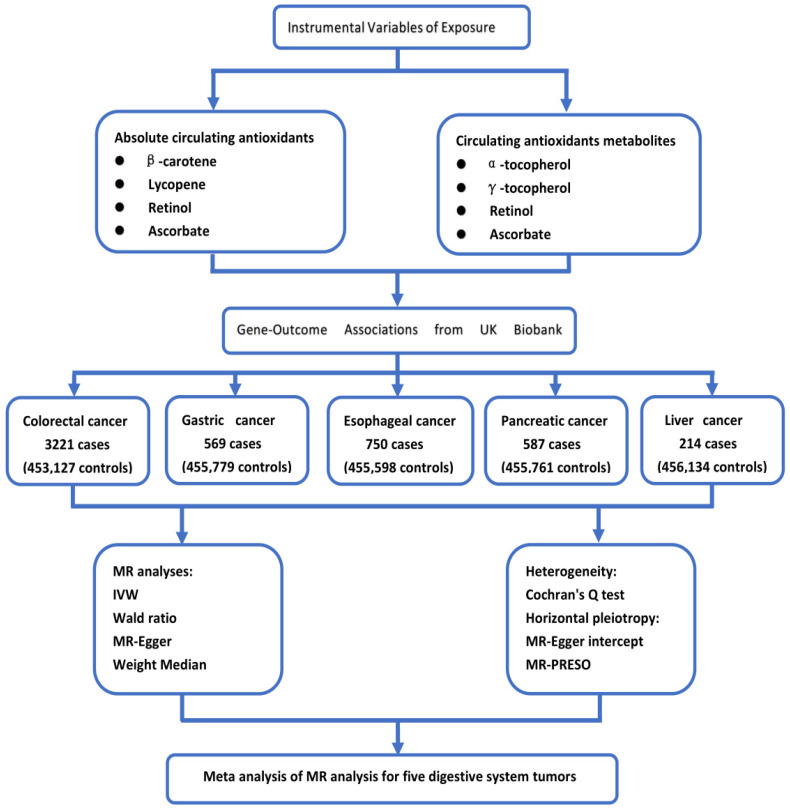
The flow chart of our study design.

The exposure factors and genetic instrumental variables of exposure selected for this study are consistent with a recent article on Mendelian randomization of antioxidants by Luo et al. [15]. For exposure factors, we chose four diet-related antioxidants: vitamin C (ascorbate), carotenoids (β-carotene, lycopene), vitamin A (retinol), and vitamin E (α-and γ-tocopherol). We analyzed absolute circulating antioxidants (true absolute levels of antioxidants in the blood) and corresponding circulating metabolites (relative concentrations of antioxidants in plasma or serum). For absolute circulating antioxidants, lycopene, retinol, β-carotene, and ascorbate were included, while for circulating metabolites of antioxidants, retinol, ascorbate, α-tocopherol, and γ-tocopherol were chosen. The flow chart of our study design is shown in Figure 1, and instrumental variables of circulating antioxidants are shown in Supplementary Table S1.

### 2.2. Absolute Circulating Antioxidants

For β-carotene, three absolute circulating antioxidant instrumental variables (*p* < 5 × 10^−8^; Linkage disequilibrium (LD) < 0.2) were identified from the Nurses’ Health Study (GWAS (Genome-wide association study), 2344 participants) [16]. Five SNPs (single-nucleotide polymorphisms) (*p* < 5 × 10^−6^; LD < 0.001) for circulating lycopene level were identified from a published GWAS study involving 441 older Amish adults [17]. We obtained summary data of two genetic variants for circulating retinol levels in a GWAS involving 5006 subjects (LD < 0.001; *p* < 5 × 10^−8^) [18]. However, only one single genetic variant associated with circulating ascorbate was identified from a meta-analysis including 5 independent studies with >15,000 participants (*p* < 2.0 × 10^−7^) [19]. Detailed information of SNPs for absolute circulating antioxidants is shown in Supplementary Table S2.

### 2.3. Circulating Antioxidant Metabolites

Genetic instrumental variables of circulating antioxidant metabolites at the suggestive genome-wide significance level were from 2 published GWAS studies based on a European population (*p* < 1 × 10^−5^) [18,20]. Finally, we identified 11 circulating antioxidant metabolites as instrumental variables of α-tocopherol (participants = 7276), 14 SNPs of ascorbate (participants = 2063), 13 genetic variants of γ-tocopherol (participants = 5822), and 24 instruments of retinol (participants = 1957). When LD > 0.001, we selected the instrumental variant with the smallest *p*-value. Detailed information of SNPs for absolute circulating antioxidants is shown in Supplementary Table S2.

### 2.4. Genetic Variants for Digestive System Tumors

GWAS summary statistics for associations of circulating levels of antioxidants with digestive system tumors (colorectal cancer, gastric cancer, esophageal cancer, pancreatic cancer, and liver cancer) were identified from the UK Biobank database [21]. Our study comprised 3221 colorectal cancer cases (453,127 controls subjects), 569 gastric cancer cases (455,779 controls subjects), 750 esophageal cancer cases (455,598 controls subjects), 587 pancreatic cancer cases (455,761 controls subjects), and 214 liver cancer cases (456,134 controls subjects).

### 2.5. Statistical Power

To evaluate the statistical power of our genetic instrumental variables, we used variance (R^2^) and F statistics. R^2^ in the Mendelian randomization study indicates the degree to which the genetic instrumental variables for antioxidants can explain the exposure [22]. R^2^ of each SNP is calculated using the following formula: $R^{2}=(\beta \times \sqrt{2\times \mathrm{MAF}\left(1-\mathrm{MAF}\right)}{)}^{2}$ (MAF: the minor allele frequency; β: the effect of the SNP on the PA.) or obtained from the original study [23]. The genetic instrumental variables for absolute circulating antioxidant explained the phenotypic variability ranging from 0.9% to 30.1%, while for circulating antioxidant metabolites, IVs explained 3.3% to 18.6%. To reduce potential weak instrumental bias, we calculated F-statistics and selected instrumental variables with F > 10 to be included in the MR analysis.

### 2.6. Statistical Analysis

All meta-analyses and MR were performed in the R-based “meta” package, “TwoSampleMR” package, and “MRPRESSO” package. (R.4.1.2, Institute for Statistics and Mathematics, Vienna, Austria)

MR applies germline genetic variation as instrumental variables to evaluate the causal relationship between a modifiable exposure or risk factor and clinically relevant outcome. Furthermore, compared with traditional methods, MR analysis can effectively avoid the influence of confounding factors and reverse causality, which benefits from the random distribution of alleles at meiosis and fixed germline genetic variation during conception [24].

We mainly assessed the causal effect of increased absolute circulating antioxidant levels or circulating antioxidant metabolite levels on the risk of different gastrointestinal cancers (colorectal cancer, gastric cancer, esophageal cancer, pancreatic cancer, and liver cancer) by inverse-variance weighted (IVW) method. For exposure where only one SNP was used as an instrumental variable, MR analysis was performed using the Wald ratio estimate method. All results were expressed as ORs (odds ratios) on digestive system tumors (colorectal cancer, gastric cancer, esophageal cancer, pancreatic cancer, and liver cancer) risk for a respective unit increment in absolute circulating antioxidants levels of ascorbate (mmol/L), lycopene (mg/dL), β-carotene (natural log-transformed levels), and retinol (natural log-transformed levels) or a 10-fold change in circulating antioxidant metabolites concentrations. In addition, we also used supplementary methods such as MR-Egger and weight median estimator for Mendelian randomized analysis. Horizontal pleiotropy occurs when a genetic variant affects the outcome variable through pathways other than or in addition to the exposure variable [25]. In the MR-Egger method, we considered the presence of an intercept and used it to assess pleiotropy. If there is a significant difference between the intercept and zero, it means that there may be horizontal pleiotropy between these genetic instrumental variables [26]. We also calculated Q statistics to measure whether there is heterogeneity between instruments. We initially used the MR-Egger intercept test to evaluate horizontal pleiotropy and further applied the MR-PRESO method to detect horizontal pleiotropy and correct for horizontal pleiotropy by excluding outlier variants [27].

Finally, we performed a meta-analysis on MR analysis results of different tumors to further evaluate the causal effect of increased antioxidant levels on the overall risk of digestive system tumors. We used the Q test and inconsistency (I^2^) test to evaluate the heterogeneity. I^2^ > 50% and *p* < 0.05 were considered to have insignificant heterogeneity [28]. If existing heterogeneity, we used a random-effects model; otherwise, a fixed-effects model was used for meta-analysis.

MR needs to meet the following assumptions: (1) instrumental variables are not related to confounding factors and outcome variables; (2) instrumental variables are associated with exposure factors; and (3) instrumental variables can only be associated with outcome variables through exposure factors [29]. Moreover, our study followed the Strengthening the Reporting of Observational Studies in Epidemiology Using Mendelian Randomization (STROBE-MR) guide [30].

## 3. Results

### 3.1. Screening of Genetic Instrumental Variables

β-carotene in absolute circulating antioxidants and γ-tocopherol in circulating antioxidant metabolites exclude one SNP (rs12934922 and rs2794327) each, as they are not available in digestive system tumors risk. Finally, two SNPs of carotene, five SNPs of lycopene, two SNPs of retinol, and one SNP of ascorbate in the absolute circulating antioxidants and eleven SNPs of α-tocopherol, twelve SNPs of γ- tocopherol, twenty-four SNPs of retinol, and fourteen SNPs of ascorbate in circulating antioxidant metabolites were included in MR analysis. Association of genome-wide SNPs for circulating antioxidants with digestive system tumors is shown in Supplementary Tables S3–S7. Furthermore, to eliminate the confounding factors related to exposure, we examined each instrumental variable one by one in PhenoScanner to make sure that the instrumental variables we included would not affect the outcome event in other ways [31].

### 3.2. Antioxidants and Colorectal Cancer

Our results showed that elevated levels of antioxidants did not reduce the risk of colorectal cancer, with consistent results observed for both absolute circulating antioxidants and circulating antioxidant metabolites. Using the IVW method, ORs for colorectal cancer per unit increase of absolute circulating antioxidants level were 0.69 (95% CI: 0.34–1.37, *p* = 0.29; β-carotene), 0.92 (95% CI: 0.73–1.16, *p* = 0.50; lycopene), 4.26 (95% CI: 0.30–61.44, *p* = 0.29; retinol), and 1.00 (95% CI: 0.95–1.05, *p* = 0.95; ascorbate), while for circulating antioxidant metabolites, ORs were 6.17 (95% CI: 0.89–42.89, *p* = 0.07; α-tocopherol), 2.17 (95% CI: 0.77–6.15, *p* = 0.15; γ-tocopherol), 1.12 (95% CI: 0.97–1.30, *p* = 0.11; retinol), and 1.00 (95% CI: 0.68–1.45, *p* = 0.98; ascorbate) (Supplementary Figure S1). When MR-Egger and weighted median methods were used to evaluate the association between antioxidants and colorectal cancer risk, the results were similar to the IVW method (Supplementary Figure S1). At the same time, Cochran’s Q test indicated that there was no significant heterogeneity between all IVs of absolute circulating antioxidants and circulating antioxidant metabolites. In addition, no obvious horizontal pleiotropic effects were observed among these SNPs by MR-Egger intercept and MR-PRESO method. The results related to heterogeneity and horizontal pleiotropy are shown in Supplementary Table S8.

### 3.3. Antioxidants and Gastric Cancer

We did not find that there was a significant causal relationship between antioxidants and gastric cancer risk using the IVW method for MR analysis. Their ORs were 0.71 (95% CI: 0.19–2.72, *p* = 0.62; β-carotene), 1.10 (95% CI: 0.70–1.73, *p* = 0.67; lycopene), 203.51 (95% CI: 0.02–2,220,283.92, *p* = 0.26; retinol), and 1.01 (95% CI: 0.92–1.11, *p* = 0.82; ascorbate) for absolute circulating antioxidants, while for circulating antioxidant metabolites, ORs were 0.24 (95% CI: 0.00–20.19, *p* = 0.53; α-tocopherol), 2.28 (95% CI: 0.15–34.71, *p* = 0.55; γ-tocopherol), 1.15 (95% CI: 0.87–1.53, *p* = 0.31; retinol), and 1.79 (95% CI: 0.91–3.50, *p* = 0.09; ascorbate) (Supplementary Figure S2). When MR analysis was performed using other methods (MR-Egger and weighted median methods), the results were consistent with the IVW method except that the MR-Egger method suggested that elevated levels of retinol in circulating antioxidant metabolites increase gastric cancer risk (OR: 2.80; 95% CI: 1.43–5.48, *p* = 0.01) (Supplementary Figure S2). Similarly, we did not observe significant heterogeneity among the instrumental variables of exposure factors through Cochran’s Q test. The MR-Egger intercept method suggested that there was no significant horizontal pleiotropy among other instrumental variables except for retinol in circulating antioxidant metabolites (intercept = −0.24, *p* = 0.01). However, when MR-PRESO analysis was subsequently applied, no significant horizontal pleiotropy was observed among all instrumental variables of exposure. The results related to heterogeneity and horizontal pleiotropy are shown in Supplementary Table S9.

### 3.4. Antioxidants and Pancreatic Cancer

Our results demonstrated no apparent causal association between increased antioxidant levels and a reduced risk of pancreatic cancer, with consistent results observed in both absolute circulating antioxidants and circulating antioxidant metabolites. The ORs of IVW method were 0.54 (95% CI: 0.26–1.11, *p* = 0.09; β-carotene), 0.91 (95% CI: 0.71–1.16, *p* = 0.43; lycopene), 0.24 (95% CI: 0.00–34.91, *p* = 0.58; retinol), and 0.99 (95% CI: 0.94–1.04, *p* = 0.74; ascorbate) for absolute circulating antioxidants, while for circulating antioxidant metabolites, ORs were 0.31 (95% CI: 0.04–2.31, *p* = 0.25; α-tocopherol), 0.79 (95% CI: 0.21–3.00, *p* = 0.72; γ-tocopherol), 0.92 (95% CI: 0.81–1.06, *p* = 0.26; retinol), and 1.22 (95% CI: 0.85–1.75, *p* = 0.28; ascorbate) (Supplementary Figure S3). Other MR analyses (MR-Egger and weighted median methods) also failed to find a causal relationship between antioxidant levels and pancreatic cancer risk (Supplementary Figure S3). Meanwhile, Cochran’s Q test did not observe significant heterogeneity between all instrumental variables of exposure. Furthermore, the MR-Egger intercept and MR-PRESO method did not find obvious horizontal pleiotropic effects among all IVs. The results related to heterogeneity and horizontal pleiotropy are shown in Supplementary Table S10.

### 3.5. Antioxidants and Liver Cancer

There is no evidence that increased levels of absolute circulating antioxidants and circulating antioxidant metabolites reduce liver cancer risk by the IVW method (absolute circulating antioxidants (OR): 1.36 (95% CI: 0.12–15.94, *p* = 0.80; β-carotene), 0.99 (95% CI: 0.49–2.01, *p* = 0.98; lycopene), 0.26 (95% CI: 0.00–99.56, *p* = 0.66; retinol), and 0.96 (95% CI: 0.86–1.07, *p* = 0.48; ascorbate); circulating antioxidant metabolites (OR): 0.20 (95% CI: 0.00–15.18, *p* = 0.47; α-tocopherol), 2.70 (95% CI: 0.16–44.54, *p* = 0.49; γ-tocopherol), 1.20 (95% CI: 0.82–1.75, *p* = 0.34; retinol), and 0.88 (95% CI: 0.34–2.27, *p* = 0.79; ascorbate)) or other MR analyses methods (MR-Egger and weighted median) (Supplementary Figure S4). Cochran’s Q test showed that there was heterogeneity among the instrumental variables of retinol in circulating antioxidant metabolites (MR Egger: Q = 35.21, *p* = 0.04; IVW: Q = 37.40, *p* = 0.03). Similarly, there was no significant horizontal pleiotropic effects among all IVs after evaluation by MR-Egger intercept and MR-PRESO methods. The results related to heterogeneity and horizontal pleiotropy are shown in Supplementary Table S11.

### 3.6. Antioxidants and Esophageal Cancer

Our results also observed no significant causal association between increased antioxidant levels and the risk of esophageal cancer by the IVW method 9absolute circulating antioxidants (OR): 2.27 (95% CI: 0.50–10.28, *p* = 0.29; β-carotene), 0.88 (95% CI: 0.58–1.33, *p* = 0.54; lycopene), 2.29 (95% CI: 0.02–280.07, *p* = 0.74; retinol), and 0.98 (95% CI: 0.90–1.08, *p* = 0.70; ascorbate); circulating antioxidant metabolites (OR): 5.58 (95% CI: 0.17–183.53, *p* = 0.33; α-tocopherol), 2.80 (95% CI: 0.40–19.89, *p* = 0.30; γ-tocopherol), 1.09 (95% CI: 0.86–1.39, *p* = 0.47; retinol), and 1.65 (95% CI: 0.88–3.07, *p* = 0.12; ascorbate)) and other MR analyses methods (MR-Egger and weighted median) (Supplementary Figure S5). Meanwhile, we found no heterogeneity among all instrumental variables (Cochran’s Q test, all *p* > 0.05) In addition, no significant horizontal pleiotropy was observed for all included IVs by MR-Egger intercept and MR-PRESO test. The results related to heterogeneity and horizontal pleiotropy are shown in Supplementary Table S12.

### 3.7. Meta-Analysis

**Figure 2:**
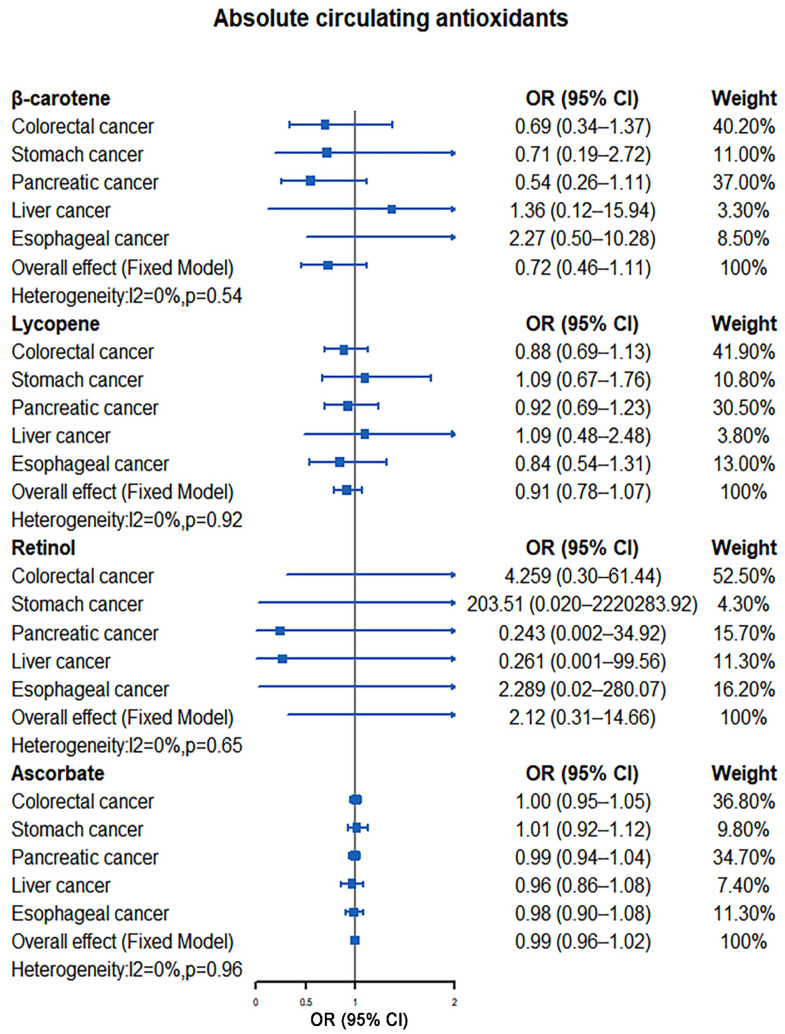
Causal association between absolute circulating antioxidants with digestive system tumors.

**Figure 3:**
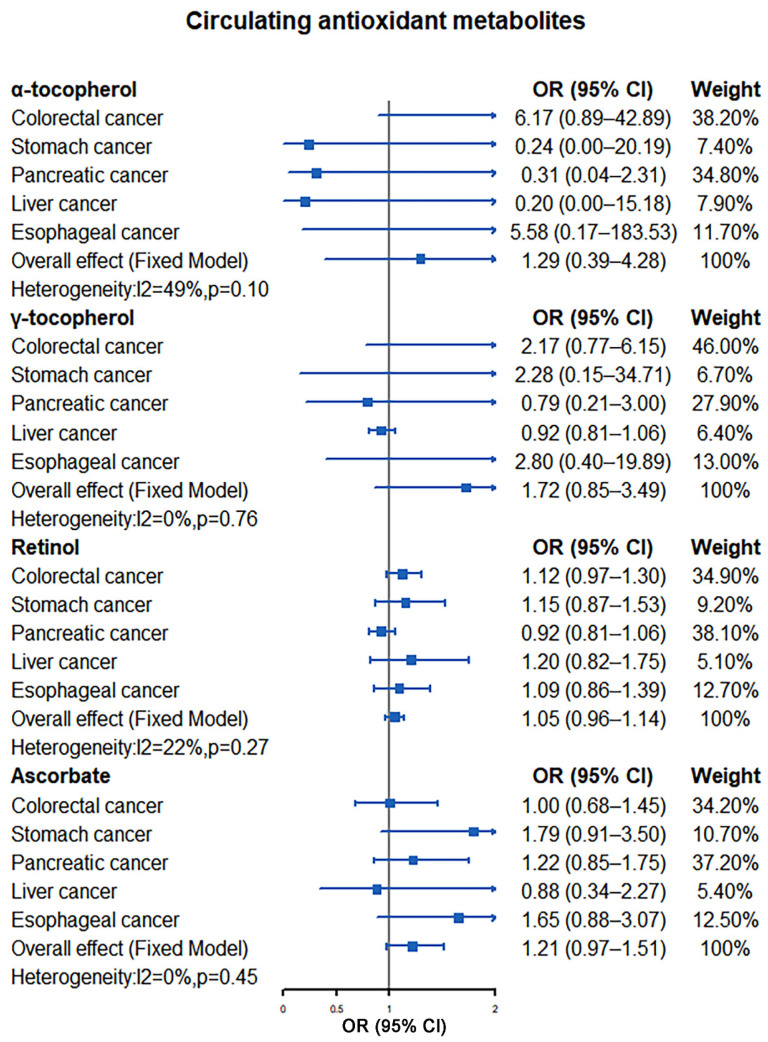
Causal association between circulating antioxidant metabolites with digestive system tumors.

Our meta-analysis results indicated that there was no significant causal relationship between the increased levels of antioxidants and the risk of digestive system tumors. For absolute circulating antioxidants, the pooled ORs are 0.72 (95% CI: 0.46–1.11, I^2^ = 0%, *p* = 0.54; β-carotene), 0.93 (95% CI: 0.81–1.08, I^2^ = 0%, *p* = 0.96; lycopene), 2.12 (95% CI: 0.31–14.66, I^2^ = 0%, *p* = 0.65; retinol), and 0.99 (95% CI: 0.96–1.02, I^2^ = 0%, *p* = 0.96; ascorbate), respectively (Figure 2). For circulating antioxidant metabolites, the pooled ORs for digestive system tumors risk per unit increase of antioxidants were 1.29 (95% CI: 0.39–4.28, I^2^ = 49%, *p* = 0.10; α-tocopherol), 1.72 (95% CI: 0.85–3.49, I^2^ = 0%, *p* = 0.76; γ-tocopherol), 1.05 (95% CI: 0.96–1.14, I^2^ = 22%, *p* = 0.27; retinol), and 1.21 (95% CI: 0.97–1.51, I^2^ = 0%, *p* = 0.45; ascorbate), respectively (Figure 3).

## 4. Discussion

In our study, we found that there was no significant causal association between the increased levels of dietary intake-related antioxidants (vitamin C (ascorbate), carotenoids (β-carotene, lycopene), vitamin A (retinol), and vitamin E (α-and γ-tocopherol)) that we included and the risk of colorectal, gastric, pancreatic, liver, and esophageal cancers. Consistent results were obtained from the absolute levels of circulating antioxidants and the levels of circulating antioxidant metabolites in the body. Meanwhile, in our meta-analysis of the results of MR analysis of different tumors, we observed little evidence that increased circulating antioxidant levels in the body reduced the risk of digestive system tumors.

Multiple observational studies have investigated the associations between diet-related antioxidants and the risk of digestive system tumors (colorectal cancer, gastric cancer, esophageal cancer, pancreatic cancer, and liver cancer). However, there has been obvious controversy over whether diet-related antioxidants reduce the risk of digestive tract tumors. Some studies have demonstrated that increased dietary intake of antioxidants can help reduce the occurrence of digestive system tumors, while some authors suggest that increased dietary intake-related antioxidants levels were not significantly associated with tumor risk. A meta-analysis by Li et al. showed that dietary intake of antioxidants (beta-carotene and vitamin A) may reduce the risk of esophageal cancer [11]. Additionally, in a recent meta-analysis by Cui et al., higher dietary vitamin E intake was associated with a lower risk of esophageal cancer [32]. For colorectal cancer, a Chinese population-based study by Luo et al. showed that a higher intake of antioxidants such as vitamin A and vitamin E could reduce the risk of colorectal cancer by 52% and 43%, respectively [12]. A meta-analysis by Dong et al. also supported that the occurrence of colorectal cancer was associated with lower serum vitamin E concentrations [33]. However, a colorectal cancer study from Fukuoka, Japan, indicated that intake of antioxidants (carotene, vitamin C, and vitamin E) was not associated with cancer risk [34]. A case-control study in Korea demonstrated that higher dietary lycopene intake may be inversely proportional to the risk of gastric cancer, and Chen et al. also believe that β-carotene has a protective effect on gastric cancer [13,35]. However, the prospective results of Anita et al. showed that the intake of vitamins and carotenoids are not significantly related to the risk of gastric cancer [36]. The association of antioxidants with pancreatic cancer and liver cancer is also controversial. Some studies support that the application of antioxidants can reduce the occurrence of liver or pancreatic cancer, while some authors believe that there is no significant correlation between tumorigenesis and antioxidant intake [37,38,39,40,41,42]. The reasons for the inconsistent results of previous studies may be that most of these studies come from case-control studies or meta-analysis and few prospective randomized studies and participants; moreover, lacking exercise, underlying diseases (inflammatory bowel disease, peptic ulcer, hepatitis B, etc.), taking drugs, alcohol consumption, smoking, and other risk factors may promote the occurrence of digestive system tumors [4]. These risk factors as one or more external factors conceal or exaggerate the relationship between exposure factors and disease, thus partially or completely distorting the real relationship between the two leading to confounding bias. At the same time, as these studies come from Asia, America, Europe, and other regions, different regions and races may also be the reason for the differences in previous research results.

Our study is the first known MR study to assess diet-related antioxidant levels and gastrointestinal tumor risk. This study has the following advantages. First, our instrumental variables were derived from the latest published articles, and we examined each instrumental variable one by one in PhenoScanner to make sure that the instrumental variables would not affect the outcome through other pathways. Moreover, we evaluated the causal association between two different sources of antioxidants (absolute circulating antioxidants and circulating antioxidant metabolites) and digestive system tumors, and the consistency of their results further supported our conclusion. Second, the relevant summary data for gastrointestinal tumors were obtained from the UKB database with a sample size of more than 400,000, which was more able to determine the causal association of genome-wide exposures and outcomes. Finally, we not only used the IVW method to detect the causal association between exposure and results but also used MR-Egger and weighted median methods to further verify. At the same time, in the horizontal pleiotropy test, we applied the MR-Egger intercept and MR-PRESO methods respectively to evaluate and correct the horizontal pleiotropy.

We must acknowledge some limitations of our study. First of all, the GWAS summary data of digestive system tumors come from a European population lacking summary data from other regions, and it is worth further exploring whether our conclusions can be generalized to the whole world. Secondly, the aggregate data of digestive system tumors we used cannot be stratified according to the covariates of interest (age, gender, smoking, drinking, underlying disease) or according to the lack of specific antioxidants in the population. As a result, it is not possible to know whether supplementation of antioxidants in certain subgroups can reduce the risk of digestive system tumors. Third, when the number of SNPs is less than three, only the Wald ratio or IVW can be used for MR, and other methods (MR Egger and weighted median) cannot be further applied to assess the association between exposure and outcome. Furthermore, horizontal pleiotropy cannot be further assessed. This may make our MR with fewer SNPs less reliable, but unfortunately, we cannot avoid it. Considering this, we chose absolute circulating antioxidants and circulating antioxidant metabolites to evaluate the content of antioxidants in vivo, and with the consistency of the two results, we tried to improve the reliability of our results.

## 5. Conclusions

In conclusion, from the perspective of genetic association, we did not find that circulating diet-related antioxidants could reduce the risk of digestive system tumors. Therefore, it is not recommended to take additional vitamin supplements to prevent the occurrence of tumors for people who are not deficient in related antioxidants.

## Data Availability

The UK Biobank data are accessible under application at https://www.ukbiobank.ac.uk/ (accessed date: 12 January 2022), and other all data described in our study are provided within this article.

## Supplementary Materials

The following supporting information can be downloaded at: https://www.mdpi.com/article/10.3390/nu14163274/s1, Figure S1: Causal association between circulating antioxidants with colorectal cancer; Figure S2: Causal association between circulating antioxidants with gastric cancer; Figure S3: Causal association between circulating antioxidants with pancreatic cancer; Figure S4: Causal association between circulating antioxidants with liver cancer; Figure S5: Causal association between circulating antioxidants with esophageal cancer; Table S1: Instrumental variables of circulating dietary-derived antioxidants; Table S2: SNPs of circulating antioxidants in the Mendelian randomization analysis; Table S3: Association of genome-wide SNPs for circulating antioxidants with colorectal cancer; Table S4: Association of genome-wide SNPs for circulating antioxidants with gastric cancer; Table S5: Association of genome-wide SNPs for circulating antioxidants with pancreatic cancer; Table S6: Association of genome-wide SNPs for circulating antioxidants with liver cancer; Table S7: Association of genome-wide SNPs for circulating antioxidants with esophageal cancer; Table S8: MR analysis results of associations between circulating antioxidants and colorectal cancer; Table S9: MR analysis results of associations between circulating antioxidants and gastric cancer; Table S10: MR analysis results of associations between circulating antioxidants and pancreatic cancer; Table S11: MR analysis results of associations between circulating antioxidants and liver cancer; Table S12: MR analysis results of associations between circulating antioxidants and esophageal cancer.
