# Supplementary material for: Diet-Derived Circulating Antioxidants and Risk of Digestive System Tumors: A Mendelian Randomization Study
Source: Nutrients. 2022 Aug 10;14(16):3274. doi: 10.3390/nu14163274 (PMC9413447; doi:10.3390/nu14163274)
Supplement: Supplementary file 1 [file nutrients-14-03274-s001.zip › nutrients-1746995-supplementary.pdf]

Supplementary Figure S1. Causal association between circulating antioxidants with colorectal cancer.

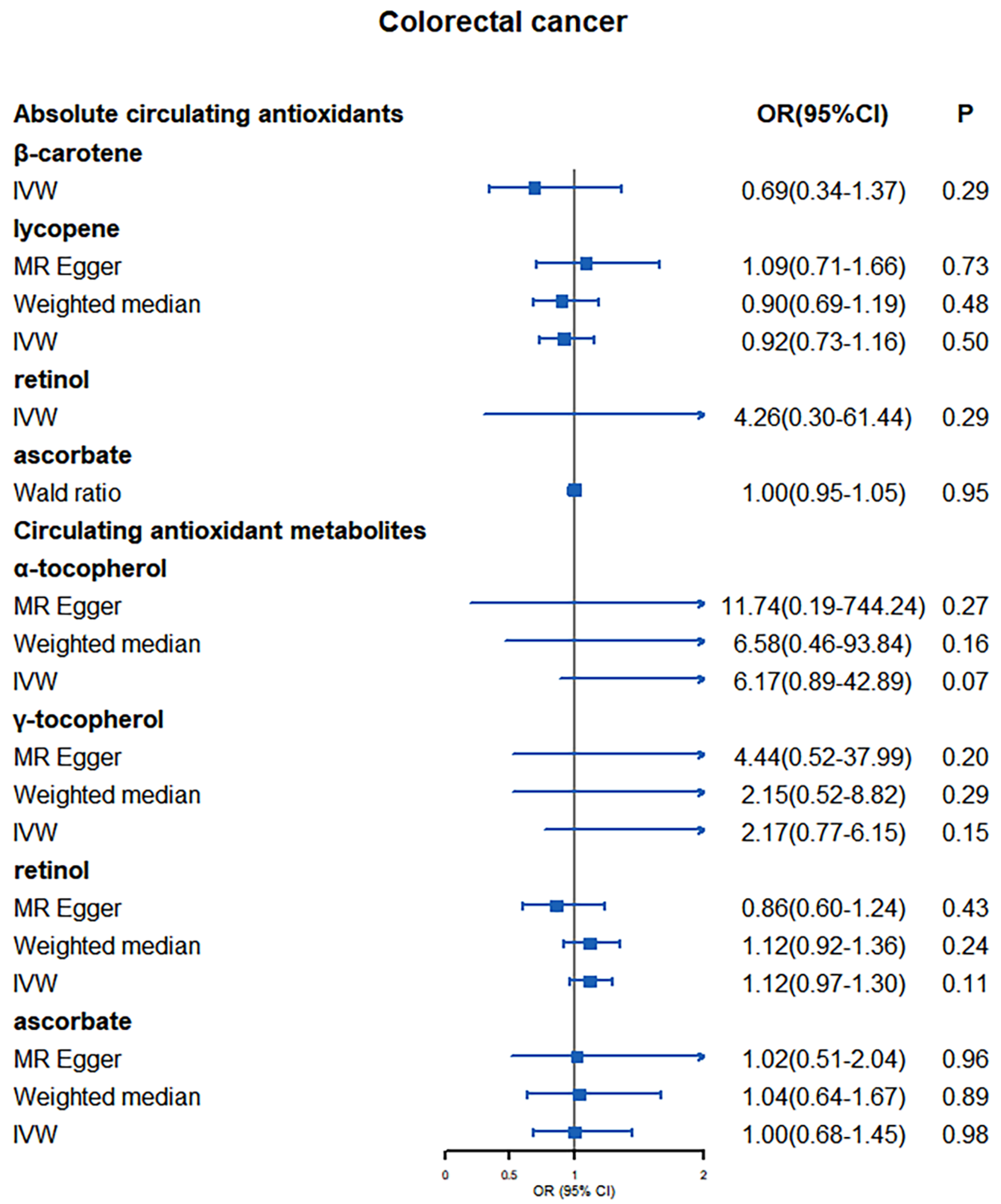

Supplementary Figure S2. Causal association between circulating antioxidants with gastric cancer.

Gastric cancer

Absolute circulating antioxidants	OR(95%CI)	P
**β-carotene**		
IVW	0.71(0.19-2.72)	0.62
**lycopene**		
MR Egger	1.80(0.78-4.13)	0.26
Weighted median	1.08(0.62-1.88)	0.80
IVW	1.10(0.70-1.73)	0.67
**retinol**		
IVW	203.51(0.02-2220283.92)	0.26
**ascorbate**		
Wald ratio	1.01(0.92-1.11)	0.82
**Circulating antioxidant metabolites**		
**α-tocopherol**		
MR Egger	38.08(0.00-401432.31)	0.46
Weighted median	0.04(0.00-8.66)	0.23
IVW	0.24(0.00-20.19)	0.53
**γ-tocopherol**		
MR Egger	31.13(0.12-8324.30)	0.26
Weighted median	2.46(0.13-45.93)	0.55
IVW	2.28(0.15-34.71)	0.55
**retinol**		
MR Egger	2.80(1.43-5.48)	0.01
Weighted median	1.12(0.78-1.61)	0.54
IVW	1.15(0.87-1.53)	0.31
**ascorbate**		
MR Egger	1.20(0.37-3.96)	0.76
Weighted median	1.72(0.63-4.73)	0.29
IVW	1.79(0.91-3.50)	0.09

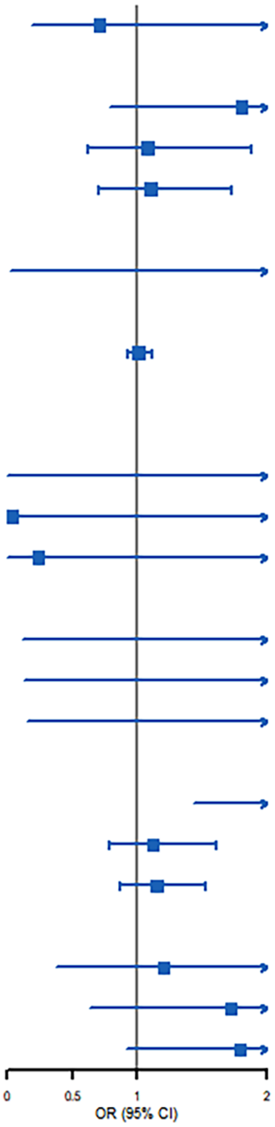

Supplementary Figure S3. Causal association between circulating antioxidants with pancreatic cancer.

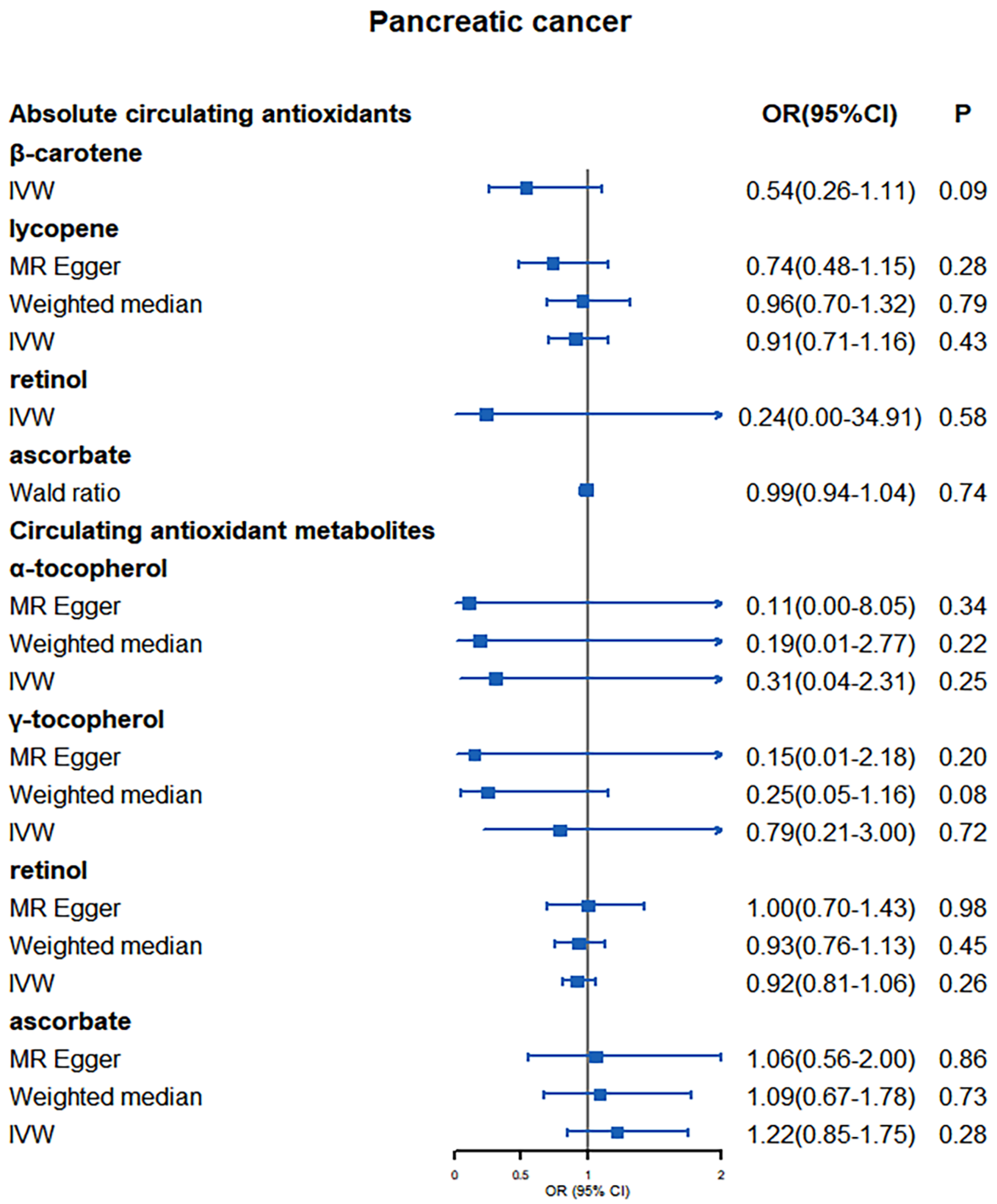

Supplementary Figure S4. Causal association between circulating antioxidants with liver cancer.

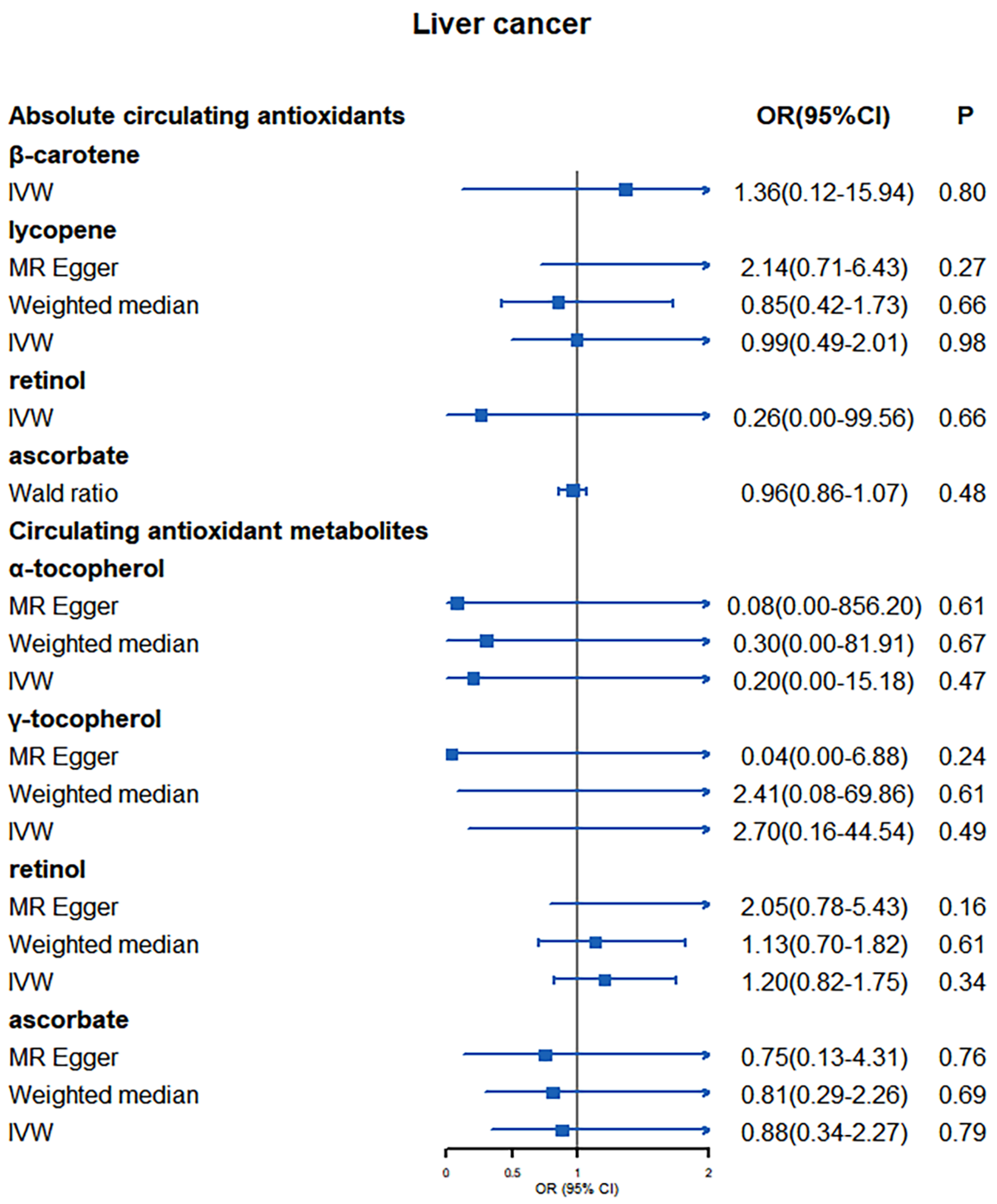

Supplementary Figure S5. Causal association between circulating antioxidants with esophageal cancer.

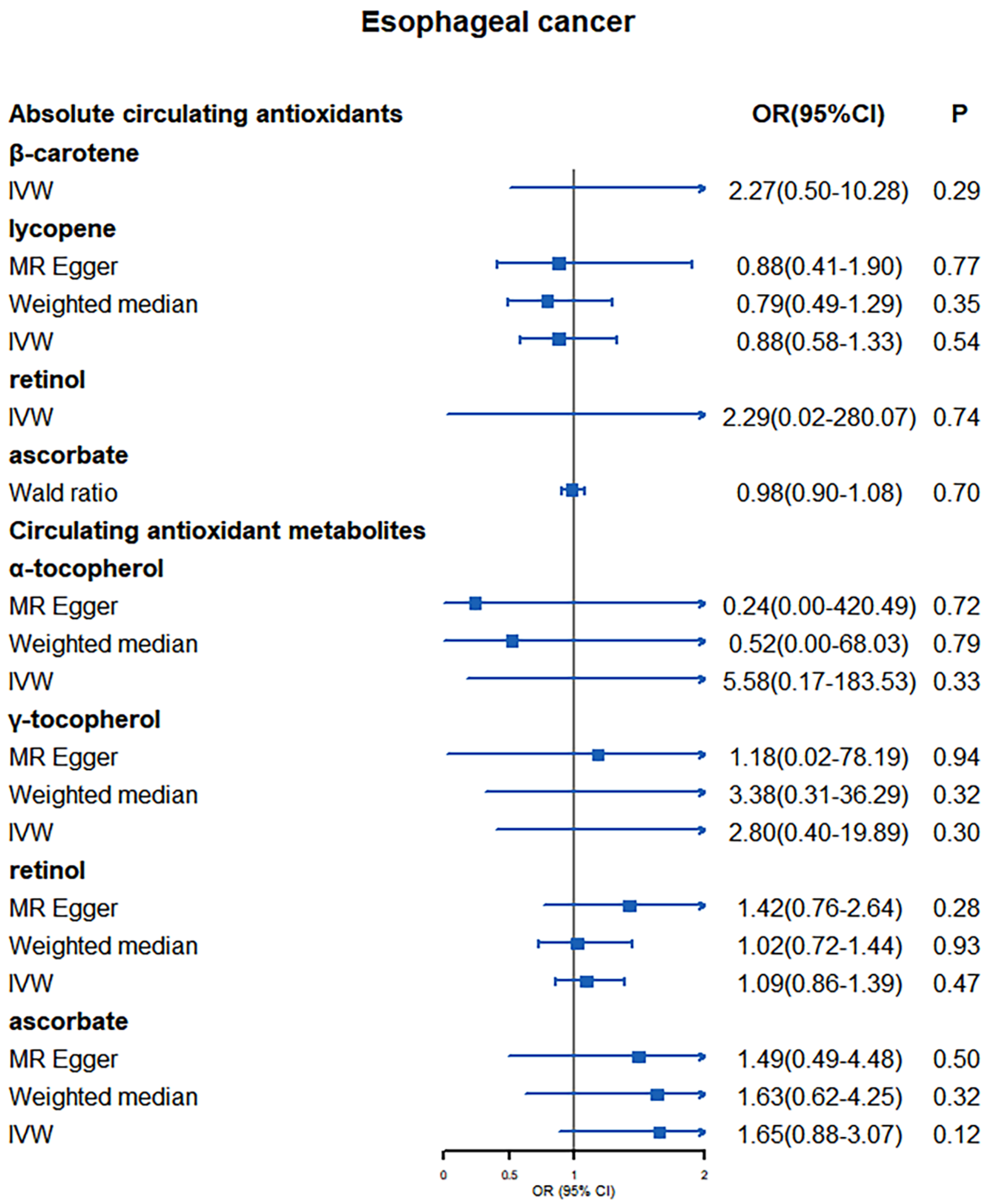

Supplementary Table S1. Instrumental variables of circulating dietary-derived antioxidants.

IVs	PMID	No. of SNPs	Study type	N	P	LD	Unit	R^2
**Absolute circulating antioxidants**								
β-carotene	23134893	5	GWAS	2,344	5.00E-08	0.2	µg/L in log-transformed	9.00%
lycopene	26861389	3	GWAS	441	5.00E-06	0.001	µg/dL	30.10%
Retinol	21878437	2	GWAS	5006	5.00E-08	0.001	µg/L in log-transformed	2.30%
Ascorbate	20519558	1	Meta-analysis	15,087	2.00E-07	0.001	µmol/L	0.90%
**Circulating antioxidants metabolites**								
α-tocopherol	24816252	11	GWAS	7,276	1.00E-05	0.001	log10-transfomed	3.30%
γ-tocopherol	24816252	13	GWAS	5,822	1.00E-05	0.001	log10-transfomed	15.00%
Retinol	28263315	24	GWAS	1,957	1.00E-05	0.001	log10-transfomed	4.80%
Ascorbate	24816252	14	GWAS	2,063	1.00E-05	0.001	log10-transfomed	18.60%

IVs, instrumental variables. LD, linkage disequilibrium.

Supplementary Table S2. SNPs of circulating antioxidants in the Mendelian randomization analysis.

SNP	Chr	Position	EA	OA	EAF	BEAT	SE	P	F-statistic	R^2
**Absolute circulating antioxidants**										
**β-carotene**										
rs12934922*	16	81301694	T	A	0.44	0.139	0.02	5.90E-10	60.21	/
rs6564851	16	81264597	G	T	0.36	0.149	0.02	1.60E-24	98.67	/
rs7501331	16	81314496	T	C	0.24	-0.067	0.02	1.60E-05	17.69	/
**lycopene**										
rs2232315	2	169757432	A	G	0.03	0.74	0.15	1.00E-06	25.01	/
rs341075	11	72257963	A	G	0.02	-0.87	0.17	6.00E-07	24.91	/
rs4635297	15	38327408	A	C	0.08	0.26	0.05	6.00E-07	25.97	/
rs7680948	4	140447105	A	C	0.2	-0.19	0.03	5.00E-09	38.52	/
rs6108801	20	10989519	C	T	0.04	-0.48	0.09	4.00E-07	27.32	/
**lycopene**										
rs10882272	10	95348182	C	T	0.35	-0.03	0.01	7.00E-15	34.57	/
rs1667255	18	29187279	C	A	0.31	0.03	0.01	6.00E-14	34.57	/
**Ascorbate**										
rs33972313	5	138715502	C	T	0.96	5.98	1.15	2.00E-07	27.14	/
**Circulating antioxidants metabolites**										
**α-tocopherol**										
rs10163969	18	9067439	T	G	0.04	-0.04	0.01	9.39E-06	19.36	0.0027
rs10245705	7	26129672	T	C	0.02	-0.07	0.01	1.95E-07	27.25	0.0037
rs10935814	3	150352873	A	G	0.1	-0.04	0.01	9.44E-06	19.87	0.0027
rs11145330	9	71733603	A	C	0.89	0.03	0.01	1.95E-06	22.7	0.0031
rs11992435	8	81526083	A	G	0.95	0.03	0.01	6.38E-06	20.63	0.0028
rs1404410	7	124248950	C	G	0.79	-0.02	0.01	4.57E-06	20.6	0.0028
rs1532701	16	55698027	A	G	0.55	0.01	0	5.07E-06	20.85	0.0029
rs2074731	22	30733914	A	C	0.17	-0.02	0	2.31E-06	22.26	0.003

rs261342	15	58731153	C	G	0.79	-0.02	0	5.41E-06	20.86	0.0029
rs7238006	18	31927898	T	C	0.93	0.03	0.01	6.77E-07	24.3	0.0033
rs7930821	11	19142695	T	C	0.02	0.07	0.01	7.53E-06	19.98	0.0027
**γ-tocopherol**										
rs10077932	5	2450679	T	C	0.14	-0.04	0.01	4.08E-06	21.35	0.0037
rs1013104	17	12187236	T	C	0.44	-0.02	0	3.83E-06	21.16	0.0036
rs10466757	12	24984990	A	T	0.16	0.06	0.01	9.56E-06	19.66	0.0034
rs10492212	12	107296945	T	C	0.16	-0.03	0.01	8.66E-06	19.53	0.0033
rs10520845	5	16879933	A	C	0.02	0.19	0.04	5.27E-06	20.78	0.0036
rs1060467	19	16024538	A	G	0.59	0.02	0	2.61E-07	26.81	0.0046
rs13336771	16	48318728	A	C	0.17	0.06	0.01	7.39E-06	20.15	0.0034
rs261301	15	58686939	T	C	0.13	0.03	0.01	2.06E-06	22.56	0.0039
rs2794327*	1	3512341	T	C	0.67	-0.04	0.01	8.78E-06	19.69	0.0034
rs5994305	22	30820707	A	G	0.83	0.03	0.01	7.15E-07	24.52	0.0042
rs6821770	4	7732805	A	G	0.14	0.04	0.01	8.92E-06	19.52	0.0033
rs7038957	9	106539439	T	C	0.83	-0.03	0.01	3.86E-06	21.43	0.0037
rs7350776	15	33689075	C	G	0.7	0.02	0.01	3.86E-06	21.12	0.0036
**Retinol**										
rs10019071	4	181442264	A	G	0.01	0.66	0.16	3.64E-06	16.72	0.0085
rs112293959	15	80358561	G	A	0.99	-0.43	0.13	5.70E-06	10.88	0.0055
rs114515641	3	118837344	G	T	0.03	0.41	0.13	7.12E-06	10.32	0.0052
rs1153379	21	16678993	A	G	0.95	-0.32	0.08	6.10E-06	14.97	0.0076
rs1176744	11	113803028	C	A	0.31	-0.21	0.05	3.52E-07	20.97	0.0106
rs118025446	6	84904002	A	G	0.04	-0.48	0.12	9.84E-06	17.38	0.0088
rs12955464	18	10141045	G	C	0.87	-0.23	0.06	3.71E-06	14.79	0.0075
rs139726207	2	223878661	G	A	0.91	0.37	0.11	4.46E-06	11.21	0.0057
rs149113848	2	81021743	G	C	0.99	-0.96	0.27	3.47E-06	13.21	0.0067
rs149478645	7	117400313	G	A	0.97	-0.51	0.14	1.30E-06	12.79	0.0065
rs17005512	4	83113756	C	G	0.17	-0.22	0.06	2.77E-06	13.33	0.0068
rs1842947	17	3316976	G	A	0.49	-0.19	0.04	8.34E-07	20.06	0.0101
rs2147337	20	1612282	G	T	0.66	0.16	0.04	9.00E-06	12.68	0.0064
rs2367816	2	33873296	G	A	0.26	0.23	0.05	9.46E-06	19.64	0.0099
rs2417325	12	14171015	T	C	0.09	0.33	0.08	1.29E-06	15.49	0.0079
rs3890033	3	195591527	C	T	0.5	0.14	0.04	8.56E-06	11.76	0.006
rs3898702	3	8011098	T	C	0.21	-0.22	0.05	3.02E-06	16.35	0.0083
rs4135385	3	41279440	G	A	0.76	0.21	0.05	9.80E-06	18.75	0.0095
rs58411567	12	124387574	A	G	0.25	-0.21	0.05	3.02E-07	15.92	0.0081
rs6550239	3	20001318	A	G	0.73	-0.18	0.05	4.40E-06	14.51	0.0074
rs75308833	5	34602282	T	C	0.97	-0.49	0.15	3.51E-06	11.27	0.0057
rs7926028	11	50213631	T	G	0.47	-0.13	0.04	2.75E-06	10.39	0.0053
rs945817	6	75454012	A	G	0.83	-0.28	0.05	6.46E-07	25.32	0.0128
rs9586119	13	88613487	C	T	0.11	0.35	0.08	3.34E-06	18.24	0.0092
**Ascorbate**										
rs11167905	5	144906846	C	T	0.14	-0.08	0.02	9.83E-07	24.03	0.0115

rs13069990	3	115620801	T	C	0.62	-0.05	0.01	4.44E-06	21.16	0.0102
rs13103690	4	9972778	G	T	0.47	0.05	0.01	5.20E-06	20.77	0.01
rs2070006	4	155513866	C	T	0.37	-0.05	0.01	4.76E-06	20.9	0.01
rs577596	22	25931606	A	G	0.38	-0.06	0.01	6.68E-07	24.74	0.0118
rs6713914	2	59523041	C	T	0.44	-0.06	0.01	3.22E-07	26.13	0.0125
rs6826474	4	96650120	T	C	0.96	-0.14	0.03	1.56E-06	23.06	0.0111
rs6834631	4	122315665	G	T	0.04	-0.13	0.03	1.03E-06	23.93	0.0115
rs7112460	11	125751859	T	C	0.93	0.11	0.02	1.14E-06	23.63	0.0113
rs8057559	16	73317172	T	C	0.96	0.14	0.03	9.10E-06	19.74	0.0095
rs808686	20	19717135	A	G	0.49	0.06	0.01	3.01E-06	21.83	0.0105
rs8105491	19	20403988	T	G	0.85	-0.07	0.01	2.30E-06	22.24	0.0107
rs9419004	10	134914522	C	G	0.28	-0.25	0.06	6.53E-06	20.35	0.0098
rs9606290	22	20218237	A	G	0.26	0.16	0.04	6.32E-06	20.4	0.0098

EA, effect allele. OA, other allele. EAF, effect allele frequency. SE, standard error. Chr, Chromsome.

*These SNPs were excluded, as they are not available in digestive system tumors risk.

Supplementary Table S3. Association of genome-wide SNPs for circulating antioxidants with colorectal cancer.

SNP	EA	OA	EAF	BETA	SE	P
**Absolute circulating antioxidants**						
**β-carotene**						
rs6564851	T	G	0.47	0.04	0.06	0.51
rs7501331	T	C	0.24	0.08	0.07	0.21
**lycopene**						
rs2232315	A	G	0.02	-0.12	0.21	0.55
rs341075	G	A	0.03	0.01	0.17	0.96
rs4635297	A	C	0.19	-0.07	0.07	0.33
rs7680948	C	A	0.27	-0.05	0.06	0.43
rs6108801	T	C	0.03	0.11	0.16	0.50
**retinol**						
rs10882272	C	T	0.38	-0.05	0.06	0.43
rs1667255	C	A	0.39	0.04	0.06	0.48
**ascorbate**						
rs33972313	T	C	0.03	-0.01	0.15	0.95
**Circulating antioxidant metabolites**						
**α-tocopherol**						
rs10163969	T	G	0.04	0.07	0.15	0.63
rs10245705	T	C	0.02	-0.43	0.21	0.05
rs10935814	A	G	0.12	-0.15	0.09	0.09
rs11145330	C	A	0.11	-0.04	0.09	0.66
rs11992435	G	A	0.06	0.12	0.12	0.32
rs1404410	G	C	0.20	0.10	0.07	0.16
rs1532701	G	A	0.46	0.02	0.06	0.69
rs2074731	A	C	0.16	-0.08	0.08	0.30

rs261342	G	C	0.22	0.01	0.07	0.85
rs7238006	C	T	0.07	-0.07	0.11	0.55
rs7930821	T	C	0.02	-0.10	0.21	0.63
**γ-tocopherol**						
rs10077932	T	C	0.18	0.08	0.07	0.25
rs1013104	T	C	0.44	0.04	0.06	0.47
rs10466757	A	T	0.25	0.07	0.06	0.31
rs10492212	T	C	0.16	-0.01	0.08	0.88
rs10520845	A	C	0.01	0.12	0.26	0.64
rs1060467	G	A	0.42	0.02	0.06	0.74
rs13336771	A	C	0.15	0.11	0.08	0.17
rs261301	T	C	0.14	0.18	0.08	0.03
rs5994305	G	A	0.17	-0.12	0.08	0.12
rs6821770	A	G	0.18	0.02	0.07	0.84
rs7038957	C	T	0.17	-0.01	0.08	0.90
rs7350776	G	C	0.30	0.03	0.06	0.65
**retinol**						
rs10019071	A	G	0.02	0.18	0.20	0.38
rs112293959	G	A	0.03	0.15	0.16	0.34
rs114515641	G	T	0.03	-0.22	0.17	0.19
rs1153379	G	A	0.06	0.09	0.13	0.46
rs1176744	C	A	0.31	-0.15	0.06	0.01
rs118025446	A	G	0.04	0.10	0.14	0.49
rs12955464	G	C	0.15	-0.03	0.08	0.72
rs139726207	G	A	0.06	-0.14	0.12	0.22
rs149113848	G	C	0.01	-0.21	0.34	0.54
rs149478645	G	A	0.02	0.22	0.19	0.24
rs17005512	C	G	0.15	0.06	0.08	0.41
rs1842947	A	G	0.47	0.04	0.06	0.53
rs2147337	T	G	0.36	0.02	0.06	0.79
rs2367816	A	G	0.24	-0.16	0.07	0.01
rs2417325	C	T	0.07	-0.06	0.11	0.60
rs3890033	C	T	0.50	0.03	0.06	0.65
rs3898702	T	C	0.20	-0.03	0.07	0.71
rs4135385	G	A	0.23	0.09	0.07	0.19
rs58411567	A	G	0.23	-0.11	0.07	0.11
rs6550239	G	A	0.23	0.05	0.07	0.48
rs75308833	T	C	0.03	-0.05	0.18	0.79
rs7926028	G	T	0.48	-0.01	0.06	0.79
rs945817	A	G	0.19	-0.02	0.07	0.76
rs9586119	C	T	0.09	0.17	0.10	0.09
**ascorbate**						
rs11167905	C	T	0.15	0.08	0.08	0.30
rs13069990	T	C	0.39	0.03	0.06	0.64

rs13103690	G	T	0.46	-0.01	0.06	0.88
rs2070006	T	C	0.38	-0.05	0.06	0.38
rs577596	A	G	0.35	-0.12	0.06	0.05
rs6713914	C	T	0.46	0.00	0.06	0.95
rs6826474	T	C	0.03	-0.24	0.17	0.17
rs6834631	G	T	0.04	0.11	0.14	0.41
rs7112460	T	C	0.06	-0.06	0.12	0.60
rs8057559	T	C	0.03	0.13	0.17	0.46
rs808686	G	A	0.49	-0.09	0.06	0.12
rs8105491	T	G	0.15	0.10	0.08	0.18
rs9419004	C	G	0.27	-0.04	0.06	0.55
rs9606290	A	G	0.26	-0.08	0.07	0.22

EA, effect allele. OA, other allele. EAF, effect allele frequency. SE, standard error.

Supplementary Table S4. Association of genome-wide SNPs for circulating antioxidants with gastric cancer.

SNP	EA	OA	EAF	BETA	SE	P
**Absolute circulating antioxidants**						
**β-carotene**						
rs6564851	T	G	0.47	0.03	0.11	0.81
rs7501331	T	C	0.24	0.10	0.13	0.44
**lycopene**						
rs2232315	A	G	0.02	-0.06	0.40	0.88
rs341075	G	A	0.03	0.42	0.32	0.19
rs4635297	A	C	0.19	-0.02	0.14	0.89
rs7680948	C	A	0.27	-0.13	0.12	0.31
rs6108801	T	C	0.03	0.08	0.31	0.79
**retinol**						
rs10882272	C	T	0.38	-0.30	0.11	0.01
rs1667255	C	A	0.39	0.02	0.11	0.87
**ascorbate**						
rs33972313	T	C	0.03	-0.07	0.30	0.82
**Circulating antioxidant metabolites**						
**α-tocopherol**						
rs10163969	T	G	0.04	-0.32	0.28	0.26
rs10245705	T	C	0.02	-0.31	0.42	0.46
rs10935814	A	G	0.12	0.14	0.17	0.42
rs11145330	C	A	0.11	-0.17	0.17	0.32
rs11992435	G	A	0.06	0.25	0.24	0.29
rs1404410	G	C	0.20	-0.12	0.14	0.40
rs1532701	G	A	0.46	0.17	0.11	0.11
rs2074731	A	C	0.16	-0.25	0.15	0.10
rs261342	G	C	0.22	-0.22	0.13	0.10
rs7238006	C	T	0.07	0.19	0.22	0.38

rs7930821	T	C	0.02	-0.23	0.40	0.57
**γ-tocopherol**						
rs10077932	T	C	0.18	-0.22	0.14	0.12
rs1013104	T	C	0.44	0.07	0.11	0.55
rs10466757	A	T	0.25	-0.01	0.13	0.91
rs10492212	T	C	0.16	0.21	0.15	0.16
rs10520845	A	C	0.01	0.26	0.51	0.60
rs1060467	G	A	0.42	-0.03	0.11	0.76
rs13336771	A	C	0.15	0.21	0.15	0.18
rs261301	T	C	0.14	0.25	0.16	0.11
rs5994305	G	A	0.17	-0.23	0.15	0.12
rs6821770	A	G	0.18	-0.05	0.14	0.72
rs7038957	C	T	0.17	-0.03	0.15	0.83
rs7350776	G	C	0.30	0.35	0.12	0.00
**retinol**						
rs10019071	A	G	0.02	-0.09	0.39	0.82
rs112293959	G	A	0.03	0.12	0.31	0.70
rs114515641	G	T	0.03	0.82	0.32	0.01
rs1153379	G	A	0.06	-0.18	0.24	0.47
rs1176744	C	A	0.31	0.05	0.12	0.70
rs118025446	A	G	0.04	-0.06	0.28	0.84
rs12955464	G	C	0.15	0.04	0.16	0.81
rs139726207	G	A	0.06	0.20	0.23	0.37
rs149113848	G	C	0.01	1.55	0.66	0.02
rs149478645	G	A	0.02	-0.75	0.37	0.04
rs17005512	C	G	0.15	-0.02	0.15	0.87
rs1842947	A	G	0.47	-0.09	0.11	0.40
rs2147337	T	G	0.36	0.11	0.11	0.32
rs2367816	A	G	0.24	-0.11	0.13	0.39
rs2417325	C	T	0.07	-0.16	0.21	0.46
rs3890033	C	T	0.50	-0.16	0.11	0.16
rs3898702	T	C	0.20	0.06	0.14	0.66
rs4135385	G	A	0.23	0.14	0.13	0.28
rs58411567	A	G	0.23	0.20	0.13	0.13
rs6550239	G	A	0.23	0.02	0.13	0.86
rs75308833	T	C	0.03	-0.44	0.34	0.20
rs7926028	G	T	0.48	0.03	0.11	0.80
rs945817	A	G	0.19	0.04	0.14	0.78
rs9586119	C	T	0.09	0.07	0.19	0.73
**ascorbate**						
rs11167905	C	T	0.15	0.08	0.15	0.61
rs13069990	T	C	0.39	-0.15	0.11	0.17
rs13103690	G	T	0.46	0.09	0.11	0.43
rs2070006	T	C	0.38	0.02	0.11	0.87

rs577596	A	G	0.35	-0.19	0.11	0.09
rs6713914	C	T	0.46	-0.01	0.11	0.94
rs6826474	T	C	0.03	0.23	0.34	0.49
rs6834631	G	T	0.04	-0.12	0.27	0.66
rs7112460	T	C	0.06	-0.16	0.22	0.48
rs8057559	T	C	0.03	-0.14	0.33	0.68
rs808686	G	A	0.49	-0.11	0.11	0.33
rs8105491	T	G	0.15	0.01	0.15	0.96
rs9419004	C	G	0.27	-0.18	0.12	0.14
rs9606290	A	G	0.26	0.04	0.13	0.78

EA, effect allele. OA, other allele. EAF, effect allele frequency. SE, standard error.

Supplementary Table S5. Association of genome-wide SNPs for circulating antioxidants with pancreatic cancer.

SNP	EA	OA	EAF	BETA	SE	P
**Absolute circulating antioxidants**						
**β-carotene**						
rs6564851	T	G	0.47	0.07	0.06	0.22
rs7501331	T	C	0.24	0.11	0.07	0.12
**lycopene**						
rs2232315	A	G	0.02	-0.39	0.21	0.07
rs341075	G	A	0.03	0.00	0.17	0.98
rs4635297	A	C	0.19	-0.02	0.07	0.82
rs7680948	C	A	0.27	0.06	0.07	0.37
rs6108801	T	C	0.03	-0.10	0.16	0.55
**retinol**						
rs10882272	C	T	0.38	0.12	0.06	0.05
rs1667255	C	A	0.39	0.03	0.06	0.58
**ascorbate**						
rs33972313	T	C	0.03	0.05	0.16	0.74
**Circulating antioxidant metabolites**						
**α-tocopherol**						
rs10163969	T	G	0.04	0.07	0.15	0.66
rs10245705	T	C	0.02	0.03	0.22	0.91
rs10935814	A	G	0.12	0.15	0.09	0.10
rs11145330	C	A	0.11	-0.03	0.09	0.78
rs11992435	G	A	0.06	0.02	0.13	0.86
rs1404410	G	C	0.20	0.00	0.07	0.96
rs1532701	G	A	0.46	-0.02	0.06	0.69
rs2074731	A	C	0.16	0.08	0.08	0.33
rs261342	G	C	0.22	-0.04	0.07	0.54
rs7238006	C	T	0.07	-0.15	0.12	0.21
rs7930821	T	C	0.02	-0.16	0.21	0.46
**γ-tocopherol**						

rs10077932	T	C	0.18	-0.09	0.08	0.23
rs1013104	T	C	0.44	-0.12	0.06	0.04
rs10466757	A	T	0.25	-0.10	0.07	0.14
rs10492212	T	C	0.16	0.04	0.08	0.61
rs10520845	A	C	0.01	0.01	0.27	0.98
rs1060467	G	A	0.42	-0.03	0.06	0.56
rs13336771	A	C	0.15	-0.09	0.08	0.25
rs261301	T	C	0.14	-0.18	0.08	0.03
rs5994305	G	A	0.17	0.05	0.08	0.50
rs6821770	A	G	0.18	0.05	0.08	0.49
rs7038957	C	T	0.17	0.10	0.08	0.22
rs7350776	G	C	0.30	-0.04	0.06	0.50
**retinol**						
rs10019071	A	G	0.02	-0.17	0.21	0.43
rs112293959	G	A	0.03	0.09	0.17	0.57
rs114515641	G	T	0.03	-0.30	0.17	0.08
rs1153379	G	A	0.06	-0.10	0.13	0.45
rs1176744	C	A	0.31	0.08	0.06	0.19
rs118025446	A	G	0.04	0.04	0.15	0.77
rs12955464	G	C	0.15	0.00	0.08	0.99
rs139726207	G	A	0.06	0.01	0.12	0.94
rs149113848	G	C	0.01	0.44	0.35	0.21
rs149478645	G	A	0.02	-0.08	0.20	0.67
rs17005512	C	G	0.15	-0.04	0.08	0.58
rs1842947	A	G	0.47	0.02	0.06	0.72
rs2147337	T	G	0.36	-0.06	0.06	0.32
rs2367816	A	G	0.24	-0.09	0.07	0.22
rs2417325	C	T	0.07	0.01	0.11	0.92
rs3890033	C	T	0.50	-0.08	0.06	0.21
rs3898702	T	C	0.20	0.04	0.07	0.56
rs4135385	G	A	0.23	-0.06	0.07	0.41
rs58411567	A	G	0.23	0.05	0.07	0.49
rs6550239	G	A	0.23	-0.03	0.07	0.62
rs75308833	T	C	0.03	-0.04	0.18	0.82
rs7926028	G	T	0.48	-0.04	0.06	0.50
rs945817	A	G	0.19	-0.01	0.07	0.93
rs9586119	C	T	0.09	-0.11	0.10	0.30
**ascorbate**						
rs11167905	C	T	0.15	-0.13	0.08	0.12
rs13069990	T	C	0.39	0.04	0.06	0.48
rs13103690	G	T	0.46	-0.01	0.06	0.86
rs2070006	T	C	0.38	0.03	0.06	0.65
rs577596	A	G	0.35	-0.04	0.06	0.52
rs6713914	C	T	0.46	-0.08	0.06	0.18

rs6826474	T	C	0.03	0.03	0.18	0.87
rs6834631	G	T	0.04	-0.02	0.14	0.88
rs7112460	T	C	0.06	-0.06	0.12	0.61
rs8057559	T	C	0.03	0.13	0.18	0.47
rs808686	G	A	0.49	0.03	0.06	0.56
rs8105491	T	G	0.15	-0.07	0.08	0.37
rs9419004	C	G	0.27	0.00	0.07	0.94
rs9606290	A	G	0.26	0.06	0.07	0.35

EA, effect allele. OA, other allele. EAF, effect allele frequency. SE, standard error.

Supplementary Table S6. Association of genome-wide SNPs for circulating antioxidants with liver cancer.

SNP	EA	OA	EAF	BETA	SE	P
**Absolute circulating antioxidants**						
**β-carotene**						
rs6564851	T	G	0.47	0.07	0.06	0.22
rs7501331	T	C	0.24	0.11	0.07	0.12
**lycopene**						
rs2232315	A	G	0.02	-0.39	0.21	0.07
rs341075	G	A	0.03	0.00	0.17	0.98
rs4635297	A	C	0.19	-0.02	0.07	0.82
rs7680948	C	A	0.27	0.06	0.07	0.37
rs6108801	T	C	0.03	-0.31	0.35	0.38
**retinol**						
rs10882272	C	T	0.38	0.12	0.06	0.05
rs1667255	C	A	0.39	0.03	0.06	0.58
**ascorbate**						
rs33972313	T	C	0.03	0.05	0.16	0.74
**Circulating antioxidant metabolites**						
**α-tocopherol**						
rs10163969	T	G	0.04	0.07	0.15	0.66
rs10245705	T	C	0.02	0.03	0.22	0.91
rs10935814	A	G	0.12	0.15	0.09	0.10
rs11145330	C	A	0.11	-0.03	0.09	0.78
rs11992435	G	A	0.06	-0.12	0.27	0.66
rs1404410	G	C	0.20	0.00	0.07	0.96
rs1532701	G	A	0.46	-0.02	0.06	0.69
rs2074731	A	C	0.16	0.08	0.08	0.33
rs261342	G	C	0.22	-0.04	0.07	0.54
rs7238006	C	T	0.07	-0.15	0.12	0.21
rs7930821	T	C	0.02	-0.16	0.21	0.46
**γ-tocopherol**						
rs10077932	T	C	0.18	-0.09	0.08	0.23
rs1013104	T	C	0.44	-0.12	0.06	0.04

rs10466757	A	T	0.25	-0.10	0.07	0.14
rs10492212	T	C	0.16	0.04	0.08	0.61
rs10520845	A	C	0.01	0.01	0.27	0.98
rs1060467	G	A	0.42	-0.03	0.06	0.56
rs13336771	A	C	0.15	-0.09	0.08	0.25
rs261301	T	C	0.14	-0.18	0.08	0.03
rs5994305	G	A	0.17	0.05	0.08	0.50
rs6821770	A	G	0.18	0.05	0.08	0.49
rs7038957	C	T	0.17	0.10	0.08	0.22
rs7350776	G	C	0.30	-0.04	0.06	0.50
**retinol**						
rs10019071	A	G	0.02	-0.17	0.21	0.43
rs112293959	G	A	0.03	0.09	0.17	0.57
rs114515641	G	T	0.03	-0.30	0.17	0.08
rs1153379	G	A	0.06	-0.10	0.13	0.45
rs1176744	C	A	0.31	0.08	0.06	0.19
rs118025446	A	G	0.04	0.04	0.15	0.77
rs12955464	G	C	0.15	0.00	0.08	0.99
rs139726207	G	A	0.06	0.01	0.12	0.94
rs149113848	G	C	0.01	0.44	0.35	0.21
rs149478645	G	A	0.02	-0.08	0.20	0.67
rs17005512	C	G	0.15	-0.04	0.08	0.58
rs1842947	A	G	0.47	0.02	0.06	0.72
rs2147337	T	G	0.36	-0.06	0.06	0.32
rs2367816	A	G	0.24	-0.09	0.07	0.22
rs2417325	C	T	0.07	0.01	0.11	0.92
rs3890033	C	T	0.50	-0.08	0.06	0.21
rs3898702	T	C	0.20	0.04	0.07	0.56
rs4135385	G	A	0.23	-0.06	0.07	0.41
rs58411567	A	G	0.23	0.05	0.07	0.49
rs6550239	G	A	0.23	-0.03	0.07	0.62
rs75308833	T	C	0.03	-0.04	0.18	0.82
rs7926028	G	T	0.48	-0.04	0.06	0.50
rs945817	A	G	0.19	-0.01	0.07	0.93
rs9586119	C	T	0.09	-0.11	0.10	0.30
**ascorbate**						
rs11167905	C	T	0.15	-0.13	0.08	0.12
rs13069990	T	C	0.39	0.04	0.06	0.48
rs13103690	G	T	0.46	-0.01	0.06	0.86
rs2070006	T	C	0.38	0.03	0.06	0.65
rs577596	A	G	0.35	-0.04	0.06	0.52
rs6713914	C	T	0.46	-0.08	0.06	0.18
rs6826474	T	C	0.03	0.03	0.18	0.87
rs6834631	G	T	0.04	-0.02	0.14	0.88

rs7112460	T	C	0.06	-0.06	0.12	0.61
rs8057559	T	C	0.03	0.13	0.18	0.47
rs808686	G	A	0.49	0.03	0.06	0.56
rs8105491	T	G	0.15	-0.07	0.08	0.37
rs9419004	C	G	0.27	0.00	0.07	0.94
rs9606290	A	G	0.26	0.06	0.07	0.35

EA, effect allele. OA, other allele. EAF, effect allele frequency. SE, standard error.

Supplementary Table S7. Association of genome-wide SNPs for circulating antioxidants with esophageal cancer.

SNP	EA	OA	EAF	BETA	SE	P
**Absolute circulating antioxidants**						
β-carotene						
rs6564851	T	G	0.47	-0.08	0.10	0.44
rs7501331	T	C	0.24	-0.19	0.12	0.11
**lycopene**						
rs2232315	A	G	0.02	0.22	0.37	0.55
rs341075	G	A	0.03	-0.33	0.30	0.27
rs4635297	A	C	0.19	-0.07	0.13	0.60
rs7680948	C	A	0.27	-0.02	0.11	0.85
rs6108801	T	C	0.03	0.09	0.28	0.75
**retinol**						
rs10882272	C	T	0.38	0.03	0.10	0.80
rs1667255	C	A	0.39	0.08	0.10	0.46
**ascorbate**						
rs33972313	T	C	0.03	0.11	0.28	0.70
**Circulating antioxidant metabolites**						
**α-tocopherol**						
rs10163969	T	G	0.04	-0.21	0.26	0.43
rs10245705	T	C	0.02	-0.26	0.39	0.50
rs10935814	A	G	0.12	0.10	0.16	0.50
rs11145330	C	A	0.11	0.02	0.16	0.92
rs11992435	G	A	0.06	0.22	0.22	0.33
rs1404410	G	C	0.20	-0.07	0.13	0.60
rs1532701	G	A	0.46	-0.08	0.10	0.43
rs2074731	A	C	0.16	-0.13	0.14	0.36
rs261342	G	C	0.22	0.17	0.12	0.17
rs7238006	C	T	0.07	-0.43	0.20	0.03
rs7930821	T	C	0.02	-0.08	0.37	0.82
**γ-tocopherol**						
rs10077932	T	C	0.18	0.14	0.13	0.29
rs1013104	T	C	0.44	-0.07	0.10	0.51
rs10466757	A	T	0.25	0.09	0.12	0.44
rs10492212	T	C	0.16	0.01	0.14	0.92

rs10520845	A	C	0.01	0.31	0.47	0.51
rs1060467	G	A	0.42	0.03	0.10	0.76
rs13336771	A	C	0.15	0.05	0.14	0.75
rs261301	T	C	0.14	0.09	0.15	0.55
rs5994305	G	A	0.17	-0.15	0.14	0.26
rs6821770	A	G	0.18	0.04	0.13	0.77
rs7038957	C	T	0.17	-0.20	0.14	0.14
rs7350776	G	C	0.30	-0.28	0.11	0.01
**retinol**						
rs10019071	A	G	0.02	0.48	0.37	0.20
rs112293959	G	A	0.03	-0.30	0.29	0.30
rs114515641	G	T	0.03	-0.16	0.30	0.60
rs1153379	G	A	0.06	-0.16	0.23	0.48
rs1176744	C	A	0.31	-0.12	0.11	0.28
rs118025446	A	G	0.04	-0.24	0.26	0.35
rs12955464	G	C	0.15	0.05	0.14	0.75
rs139726207	G	A	0.06	0.16	0.21	0.45
rs149113848	G	C	0.01	-0.62	0.61	0.31
rs149478645	G	A	0.02	-0.31	0.34	0.36
rs17005512	C	G	0.15	-0.29	0.14	0.04
rs1842947	A	G	0.47	-0.01	0.10	0.93
rs2147337	T	G	0.36	0.03	0.11	0.80
rs2367816	A	G	0.24	-0.04	0.12	0.73
rs2417325	C	T	0.07	-0.01	0.20	0.97
rs3890033	C	T	0.50	0.06	0.11	0.55
rs3898702	T	C	0.20	0.24	0.13	0.06
rs4135385	G	A	0.23	-0.03	0.12	0.79
rs58411567	A	G	0.23	0.03	0.12	0.79
rs6550239	G	A	0.23	-0.23	0.12	0.05
rs75308833	T	C	0.03	0.12	0.32	0.70
rs7926028	G	T	0.48	0.00	0.10	0.99
rs945817	A	G	0.19	0.04	0.13	0.73
rs9586119	C	T	0.09	0.24	0.18	0.19
**ascorbate**						
rs11167905	C	T	0.15	-0.15	0.14	0.28
rs13069990	T	C	0.39	0.07	0.10	0.47
rs13103690	G	T	0.46	0.01	0.10	0.92
rs2070006	T	C	0.38	0.04	0.10	0.72
rs577596	A	G	0.35	0.06	0.11	0.60
rs6713914	C	T	0.46	-0.15	0.10	0.13
rs6826474	T	C	0.03	-0.24	0.31	0.45
rs6834631	G	T	0.04	0.04	0.25	0.86
rs7112460	T	C	0.06	-0.28	0.21	0.17
rs8057559	T	C	0.03	-0.07	0.31	0.81

rs808686	G	A	0.49	-0.15	0.10	0.13
rs8105491	T	G	0.15	-0.11	0.14	0.45
rs9419004	C	G	0.27	-0.17	0.11	0.14
rs9606290	A	G	0.26	0.00	0.12	0.99

EA, effect allele. OA, other allele. EAF, effect allele frequency. SE, standard error.

Supplementary Table S8. MR analysis results of associations between circulating antioxidants and colorectal cancer.

	SE	β(95%CI)	OR(95%CI)	P	Heterogeneity				pleiotropy			
**Absolute circulating antioxidants**					MR Egger		IVW		MR-Egger intercept		MR-PRESO	
**β-carotene**					Q	P	Q	P	intercept	P	No. of outlier	P
MR Egger	/	/	/	/								
Weighted median	/	/	/	/	/	/	0.86	0.35	/	/	/	/
IVW	0.35	-0.37(-1.07-0.32)	0.69(0.34-1.37)	0.29								
**lycopene**												
MR Egger	0.22	0.08(-0.35-0.51)	1.09(0.71-1.66)	0.73								
Weighted median	0.14	-0.10(-0.38-0.18)	0.90(0.69-1.19)	0.48	1.17	0.76	1.95	0.75	-0.07	0.44	NA	0.78
IVW	0.12	-0.08(-0.31-0.15)	0.92(0.73-1.16)	0.50								
**retinol**												
MR Egger	/	/	/	/								
Weighted median	/	/	/	/	/	/	0.00	0.95	/	/	/	/
IVW	1.36	1.45(-1.22-4.12)	4.26(0.30-61.44)	0.29								
**ascorbate**												
Wald ratio	0.03	0.00(-0.05-0.05)	1.00(0.95-1.05)	0.95	/	/	/	/	/	/	/	/
**Circulating antioxidant metabolites**												
**α-tocopherol**												
MR Egger	2.12	2.46(-1.69-6.61)	11.74(0.19-744.24)	0.27								
Weighted median	1.36	1.88(-0.77-4.54)	6.58(0.46-93.84)	0.16	8.60	0.47	8.72	0.56	-0.02	0.74	NA	0.71
IVW	0.99	1.82(-0.12-3.76)	6.17(0.89-42.89)	0.07								
**γ-tocopherol**												
MR Egger	1.10	1.49(-0.66-3.64)	4.44(0.52-37.99)	0.20								
Weighted median	0.72	0.76(-0.65-2.18)	2.15(0.52-8.82)	0.29	9.98	0.44	10.54	0.48	-0.03	0.47	NA	0.53
IVW	0.53	0.77(-0.27-1.82)	2.17(0.77-6.15)	0.15								
**retinol**												
MR Egger	0.19	-0.15(-0.51-0.21)	0.86(0.60-1.24)	0.43								
Weighted median	0.10	0.11(-0.08-0.31)	1.12(0.92-1.36)	0.24	24.44	0.32	27.10	0.25	0.07	0.14	NA	0.27
IVW	0.07	0.12(-0.03-0.26)	1.12(0.97-1.30)	0.11								
**ascorbate**												
MR Egger	0.35	0.02(-0.67-0.71)	1.02(0.51-2.04)	0.96								
Weighted median	0.24	0.04(-0.44-0.52)	1.04(0.64-1.67)	0.89	15.38	0.22	15.38	0.28	0.00	0.94	NA	0.35
IVW	0.19	0.00(-0.38-0.37)	1.00(0.68-1.45)	0.98								

SE, standard error. OR, odds ratio. IVW, Inverse variance weighted.

Supplementary Table S9. MR analysis results of associations between circulating antioxidants and gastric cancer.

	SE	β(95%CI)	OR(95%CI)	P	Heterogeneity				Pleiotropy			
**Absolute circulating antioxidants**					MR Egger		IVW		MR-Egger intercept		MR-PRESO	
β-carotene					Q	P	Q	P	intercept	P	No. of outlier	P
MR Egger	/	/	/	/								
Weighted median	/	/	/	/	/	/	0.40	0.52	/	/	/	/
IVW	0.69	-0.34(-1.68-1.00)	0.71(0.19-2.72)	0.62								
**lycopene**												
MR Egger	0.42	0.59(-0.24-1.42)	1.80(0.78-4.13)	0.26								
Weighted median	0.28	0.07(-0.48-0.63)	1.08(0.62-1.88)	0.80	0.75	0.86	2.65	0.62	-0.21	0.26	NA	0.50
IVW	0.23	0.10(-0.35-0.55)	1.10(0.70-1.73)	0.67								
**retinol**												
MR Egger	/	/	/	/								
Weighted median	/	/	/	/	/	/	3.21	0.07	/	/	/	/
IVW	4.74	5.32(-3.98-14.61)	203.51(0.02-2220283.92)	0.26								
**ascorbate**												
Wald ratio	0.05	0.01(-0.09-0.11)	1.01(0.92-1.11)	0.82	/	/	/	/	/	/	/	/
**Circulating antioxidant metabolites**												
**α-tocopherol**												
MR Egger	4.73	3.64(-5.62-12.9)	38.08(0.00-401432.31)	0.46								
Weighted median	2.81	-3.34(-8.84-2.16)	0.04(0.00-8.66)	0.23	11.85	0.22	13.78	0.18	-0.15	0.26	NA	0.19
IVW	2.26	-1.42(-5.84-3.01)	0.24(0.00-20.19)	0.53								
**γ-tocopherol**												
MR Egger	2.85	3.44(-2.15-9.03)	31.13(0.12-8324.30)	0.26								
Weighted median	1.49	0.90(-2.03-3.83)	2.46(0.13-45.93)	0.55	17.81	0.06	19.76	0.06	-0.12	0.32	NA	0.09
IVW	1.39	0.83(-1.89-3.55)	2.28(0.15-34.71)	0.55								
**retinol**												
MR Egger	0.34	1.03(0.36-1.70)	2.80(1.43-5.48)	0.01								
Weighted median	0.19	0.11(-0.25-0.48)	1.12(0.78-1.61)	0.54	19.31	0.63	27.19	0.25	-0.24	0.01	NA	0.25
IVW	0.14	0.14(-0.14-0.42)	1.15(0.87-1.53)	0.31								
**ascorbate**												
MR Egger	0.61	0.19(-1.00-1.38)	1.20(0.37-3.96)	0.76								
Weighted median	0.51	0.54(-0.46-1.55)	1.72(0.63-4.73)	0.29	6.58	0.88	7.21	0.89	0.05	0.44	NA	0.92
IVW	0.34	0.58(-0.09-1.25)	1.79(0.91-3.50)	0.09								

SE, standard error. OR, odds ratio. IVW, Inverse variance weighted.

Supplementary Table S10. MR analysis results of associations between circulating antioxidants and pancreatic cancer.

	SE	β(95%CI)	OR(95%CI)	P	Heterogeneity				pleiotropy			
**Absolute circulating antioxidants**					MR Egger		IVW		MR-Egger intercept		MR-PRESO	
**β-carotene**					Q	P	Q	P	intercept	P	No. of outlier	P
MR Egger		/	/	/								
Weighted median		/	/	/	/	/	1.03	0.31	/	/	/	/
IVW	0.37	-0.62(-1.36-0.11)	0.54(0.26-1.11)	0.09								
**lycopene**												
MR Egger	0.23	-0.30(-0.74-0.14)	0.74(0.48-1.15)	0.28								
Weighted median	0.16	-0.04(-0.36-0.27)	0.96(0.70-1.32)	0.79	2.79	0.43	3.94	0.41	0.09	0.46	NA	0.40
IVW	0.12	-0.10(-0.34-0.14)	0.91(0.71-1.16)	0.43								
**retinol**												
MR Egger	/	/	/	/								
Weighted median	/	/	/	/	/	/	3.20	0.07	/	/	/	/
IVW	2.53	-1.41(-6.38-3.55)	0.24(0.00-34.91)	0.58								
**ascorbate**												
Wald ratio	0.03	-0.01(-0.06-0.04)	0.99(0.94-1.04)	0.74	/	/	/	/	/	/	/	/
**Circulating antioxidant metabolites**												
**α-tocopherol**												
MR Egger	2.20	-2.23(-6.55-2.09)	0.11(0.00-8.05)	0.34								
Weighted median	1.37	-1.66(-4.34-1.02)	0.19(0.01-2.77)	0.22	5.04	0.83	5.34	0.87	0.03	0.60	NA	0.79
IVW	1.03	-1.18(-3.19-0.84)	0.31(0.04-2.31)	0.25								
**γ-tocopherol**												
MR Egger	1.36	-1.88(-4.53-0.78)	0.15(0.01-2.18)	0.20								
Weighted median	0.79	-1.39(-2.93-0.15)	0.25(0.05-1.16)	0.08	14.09	0.17	16.77	0.11	0.07	0.20	NA	0.14
IVW	0.68	-0.24(-1.58-1.10)	0.79(0.21-3.00)	0.72								
**retinol**												
MR Egger	0.18	0.00(-0.36-0.35)	1.00(0.70-1.43)	0.98								
Weighted median	0.10	-0.08(-0.27-0.12)	0.93(0.76-1.13)	0.45	14.67	0.88	14.86	0.90	-0.02	0.66	NA	0.90
IVW	0.07	-0.08(-0.22-0.06)	0.92(0.81-1.06)	0.26								
**ascorbate**												
MR Egger	0.33	0.06(-0.58-0.70)	1.06(0.56-2.00)	0.86								
Weighted median	0.25	0.09(-0.40-0.58)	1.09(0.67-1.78)	0.73	6.81	0.87	7.09	0.90	0.02	0.61	NA	0.90
IVW	0.18	0.20(-0.16-0.56)	1.22(0.85-1.75)	0.28								

SE, standard error. OR, odds ratio. IVW, Inverse variance weighted.

Supplementary Table S11. MR analysis results of associations between circulating antioxidants and liver cancer.

	SE	β(95%CI)	OR(95%CI)	P	Heterogeneity				pleiotropy			
**Absolute circulating antioxidants**					MR Egger		IVW		MR-Egger intercept		MR-PRESO	
**β-carotene**					Q	P	Q	P	intercept	P	No. of outlier	P
MR Egger												
Weighted median					/	/	2.55	0.11	/	/	/	/
IVW	1.25	0.31(-2.15-2.77)	1.36(0.12-15.94)	0.80								
**lycopene**												
MR Egger	0.56	0.76(-0.34-1.86)	2.14(0.71-6.43)	0.27								
Weighted median	0.36	-0.16(-0.87-0.55)	0.85(0.42-1.73)	0.66	3.98	0.26	7.52	0.11	-0.33	0.20	NA	0.17
IVW	0.36	-0.01(-0.72-0.70)	0.99(0.49-2.01)	0.98								
**retinol**												
MR Egger												
Weighted median					/	/	0.03	0.86	/	/	/	/
IVW	3.03	-1.34(-7.28-4.60)	0.26(0.00-99.56)	0.66								
**ascorbate**												
Wald ratio	0.06	-0.04(-0.15-0.07)	0.96(0.86-1.07)	0.48	/	/	/	/	/	/	/	/
**Circulating antioxidant metabolites**												
**α-tocopherol**												
MR Egger	4.71	-2.47(-11.7-6.75)	0.08(0.00-856.20)	0.61								
Weighted median	2.87	-1.22(-6.84-4.41)	0.30(0.00-81.91)	0.67	6.96	0.64	7.01	0.72	0.03	0.84	NA	0.70
IVW	2.20	-1.59(-5.9-2.72)	0.20(0.00-15.18)	0.47								
**γ-tocopherol**												
MR Egger	2.69	-3.35(-8.63-1.93)	0.04(0.00-6.88)	0.24								
Weighted median	1.72	0.88(-2.49-4.25)	2.41(0.08-69.86)	0.61	11.86	0.29	15.87	0.15	0.19	0.10	NA	0.13
IVW	1.43	0.99(-1.81-3.80)	2.70(0.16-44.54)	0.49								
**retinol**												
MR Egger	0.50	0.72(-0.25-1.69)	2.05(0.78-5.43)	0.16								
Weighted median	0.24	0.12(-0.35-0.60)	1.13(0.70-1.82)	0.61	35.21	0.04	37.40	0.03	-0.14	0.25	NA	0.19
IVW	0.19	0.18(-0.19-0.56)	1.20(0.82-1.75)	0.34								
**ascorbate**												
MR Egger	0.89	-0.28(-2.03-1.46)	0.75(0.13-4.31)	0.76								
Weighted median	0.52	-0.21(-1.24-0.82)	0.81(0.29-2.26)	0.69	19.63	0.07	19.71	0.10	0.02	0.84	NA	0.19
IVW	0.48	-0.13(-1.07-0.82)	0.88(0.34-2.27)	0.79								

SE, standard error. OR, odds ratio. IVW, Inverse variance weighted.

Supplementary Table S12. MR analysis results of associations between circulating antioxidants and esophageal cancer.

	SE	β(95%CI)	OR(95%CI)	P	Heterogeneity				pleiotropy			
**Absolute circulating antioxidants**					MR Egger		IVW		MR-Egger intercept		MR-PRESO	
**β-carotene**					Q	P	Q	P	intercept	P	No. of outlier	P
MR Egger	/	/	/	/								
Weighted median	/	/	/	/	/	/	1.47	0.22	/	/	/	/
IVW	0.77	0.82(-0.69-2.33)	2.27(0.50-10.28)	0.29								
**lycopene**												
MR Egger	0.39	-0.13(-0.90-0.64)	0.88(0.41-1.90)	0.77								
Weighted median	0.25	-0.23(-0.72-0.26)	0.79(0.49-1.29)	0.35	1.60	0.66	1.60	0.81	0.00	1.00	NA	0.72
IVW	0.21	-0.13(-0.55-0.29)	0.88(0.58-1.33)	0.54								
**retinol**												
MR Egger	/	/	/	/								
Weighted median	/	/	/	/	/	/	0.49	0.48	/	/	/	/
IVW	2.45	0.83(-3.98-5.64)	2.29(0.02-280.07)	0.74								
**ascorbate**												
Wald ratio	0.05	-0.02(-0.11-0.07)	0.98(0.90-1.08)	0.70	/	/	/	/	/	/	/	/
**Circulating antioxidant metabolites**												
**α-tocopherol**												
MR Egger	3.81	-1.44(-8.91-6.04)	0.24(0.00-420.49)	0.72								
Weighted median	2.49	-0.66(-5.54-4.22)	0.52(0.00-68.03)	0.79	8.86	0.45	9.73	0.46	0.10	0.37	NA	0.51
IVW	1.78	1.72(-1.77-5.21)	5.58(0.17-183.53)	0.33								
**γ-tocopherol**												
MR Egger	2.14	0.16(-4.04-4.36)	1.18(0.02-78.19)	0.94								
Weighted median	1.21	1.22(-1.16-3.59)	3.38(0.31-36.29)	0.32	11.71	0.30	11.96	0.37	0.04	0.65	NA	0.44
IVW	1.00	1.03(-0.93-2.99)	2.80(0.40-19.89)	0.30								
**retinol**												
MR Egger	0.32	0.35(-0.27-0.97)	1.42(0.76-2.64)	0.28								
Weighted median	0.18	0.02(-0.33-0.37)	1.02(0.72-1.44)	0.93	21.15	0.51	21.93	0.52	-0.07	0.39	NA	0.54
IVW	0.12	0.09(-0.15-0.33)	1.09(0.86-1.39)	0.47								
**ascorbate**												
MR Egger	0.56	0.40(-0.71-1.5)	1.49(0.49-4.48)	0.50								
Weighted median	0.49	0.49(-0.47-1.45)	1.63(0.62-4.25)	0.32	9.28	0.68	9.33	0.75	0.01	0.83	NA	0.79
IVW	0.32	0.50(-0.12-1.12)	1.65(0.88-3.07)	0.12								

SE, standard error. OR, odds ratio. IVW, Inverse variance weighted.
